# Variability of Oral and Pharyngeal Transit Between Two Consecutive Swallows in Chagas’ Disease

**DOI:** 10.4021/gr574w

**Published:** 2013-09-09

**Authors:** Roberto Oliveira Dantas, Carla Manfredi dos Santos, Rachel de Aguiar Cassiani, Weslania Viviane do Nascimento

**Affiliations:** a Department of Medicine of the Medical School of Ribeirao Preto, University of Sao Paulo, Ribeirao Preto SP, Brazil

**Keywords:** Chagas’ disease, Swallowing, Deglutition, Swallowing control, Megaesophagus, Pharynx

## Abstract

**Background:**

Chagas’ disease causes dysphagia, regurgitation and retention of food in the esophageal body. Patients have longer pharyngeal clearance, which might be consequent of the involvement of the central nervous system or an adaptation to the esophageal transit impairment. If there is central nervous system involvement by the disease, we expect a larger difference in the oral and pharyngeal phases of swallowing between two consecutive swallows than that seen in controls. Our objective was to evaluate the difference of oral and pharyngeal transit duration between two consecutive swallows in patients with Chagas’ disease compared with controls.

**Methods:**

By videofluoroscopy, the duration of oral and pharyngeal transit, pharyngeal clearance, upper esophageal sphincter transit, hyoid movement and oropharyngeal transit was measured in 17 patients with Chagas’ disease and 15 asymptomatic volunteers. Each subject swallowed in duplicate and in sequence 5 mL and 10 mL of barium liquid and 5 mL and 10 mL of barium paste boluses. The differences were calculated between the two swallows of each volume and consistency in patients and controls.

**Results:**

There were no differences between controls and patients in the values of the differences between the two consecutive swallows, except for the hyoid movement duration of the 5 mL liquid bolus, causing a higher difference in controls than in patients.

**Conclusion:**

Oral and pharyngeal transit variation between two consecutive swallows is similar between patients with Chagas’ disease and controls, which suggests that the longer pharyngeal clearance duration previously described is not a consequence of impairment of the central nervous system control of swallowing.

## Introduction

The esophageal involvement by Chagas’ disease has as consequence megaesophagus, aperistaltic contraction in the esophageal body and partial or absent relaxation of the lower esophageal sphincter [1-3]. Esophageal dismotility and the impairment of esophageal bolus transit are considered to be responsible for the most frequent symptoms: dysphagia and regurgitation.

However, there is also alteration of pharyngeal clearance [4-6]. Chagas’ disease patients have a longer pharyngeal clearance with swallows of a 10 mL liquid bolus [5] and 10 mL paste bolus [4, 6] when compared with asymptomatic healthy subjects. The alteration of pharyngeal clearance may be an adaptation to difficulty in esophageal transit associated with bolus retention inside the esophageal body, or the involvement of the central control of swallowing. However, it is not likely that alteration of the pharyngeal phase of swallowing is consequent of central nervous system (CNS) involvement because clinical, electroencephalographic and magnetic resonance imaging evaluation of the CNS in the disease does not demonstrate neurological dysfunction that could explain pharyngeal alterations [7, 8].

Our hypothesis is that the increase in the pharyngeal clearance duration described in Chagas’ disease patients with esophageal involvement is an adaptation to the impairment of esophageal bolus transit. If the hypothesis is true, we expect that two consecutive swallows have similar variation compared with that seen in healthy subjects, a demonstration that there is no impairment of the central control of swallowing. The objective of this investigation was to evaluate the variation in oral and pharyngeal transit durations between two consecutive swallows of two volumes of liquid and paste boluses.

## Material and Methods

We studied 17 patients with Chagas’ disease and 15 asymptomatic volunteers who participated in a previous study [6]. The group of Chagas’ disease patients consisted of 9 women and 8 men, aged 31 - 67 years, mean 53.3 years, with dysphagia and a positive serologic examination for Chagas’ disease. Esophageal involvement by the disease was diagnosed by contrast radiography, which showed esophageal retention of 100% barium sulfate for more than 30 seconds after ingestion of a volume of 100 mL, with an increase in distal esophageal diameter (higher than 4 cm) in five patients. The control group consisted of asymptomatic healthy volunteers who had never lived in endemic areas for Chagas’ disease, 7 women and 8 men, aged 35 - 69 years, mean 55.2 years. Subjects with heart diseases, diabetes, hypertension, respiratory, neurological, or renal diseases, or those who were taking drugs were excluded from both groups. No subject had been previously treated for esophageal or gastric diseases.

The study was conducted at the University Hospital of the Medical School of Ribeirao Preto, University of Sao Paulo, and the protocol of the investigation was approved by the Human Research Committee of the University Hospital of Ribeirao Preto. Written informed consent was obtained from each participant.

Swallowing was assessed by videofluoroscopy. The equipment used for radiological examination was the Arcomax angiograph unit (Phillips, model BV 300, Veenpluis, The Netherlands) The images were recorded at 60 frames per second using the digital processing system Ever Focus model EDSR 100 V1.2 (Taipei, Taiwan) with a DVR monitor (Ever Focus) and a digital clock that indicates time in minutes, seconds and the number of frames on each video frame. Mouth, pharynx and proximal esophagus were imaged in lateral projection, with the subjects sitting in a chair and both feet on the ground. Boluses of 5 mL and 10 mL of a liquid and 5 mL and 10 mL of a paste were swallowed in duplicate. For the liquid bolus, barium sulfate (Bariogel^®^ 100%, Laboratory Cristalia, Itapira, SP, Brazil) was offered with the aid of a spoon. For the paste bolus, we added 30 mL of 100% liquid barium sulfate to 3 g of the food thickener Nutilis (Nutricia Cuyk B.V., DJ Cuyk, The Netherlands), which was also offered with a spoon.

The following features were timed: 1). onset of propulsive tongue tip movement at the maxillary incisors; 2). onset and end of hyoid movement; 3). passage of the bolus head through the fauces; 4). passage of the bolus tail through the fauces; 5). onset and offset of upper esophageal sphincter (UES) opening. From these timings, we calculated the oral transit (tongue tip at incisors to passage of the bolus tail through the fauces), pharyngeal transit (bolus tail at fauces to offset of UES opening), pharyngeal clearance (bolus head at fauces to offset of UES opening), UES opening duration (time between onset and offset of UES opening), duration of hyoid movement (time between onset and end of hyoid movement), and oropharyngeal transit (tongue tip at incisors to offset of UES opening). In this investigation we calculated the difference between the two consecutive swallows of the same bolus volume and the same bolus consistency, to evaluate the variation between the two consecutive swallows. The swallows were performed in the sequence: two 5 mL liquid, two 10 mL liquid, two 5 mL paste, two 10 mL paste, with an interval of 30 - 60 seconds between the two swallows.

Statistical analysis was done by the Mann-Whitney test. The results are shown, in milliseconds (ms), as mean, standard deviation (SD) and median. A P value < 0.05 was considered significant.

## Results

There was no difference between controls and patients in the values of the difference between the consecutive swallows in the duration of oral transit, pharyngeal transit, pharyngeal clearance, UES opening, hyoid movement and oropharyngeal transit, with swallows of liquid (Table 1) and paste (Table 2) boluses. One exception found was for the hyoid movement duration with swallows of the 5 mL liquid bolus, which has a median in variation of consecutive swallows of 167 ms for controls and 67 ms for patients (P = 0.02).

**Table 1:** Differences, in Milliseconds, Between Two Consecutive Swallows of 5 mL and 10 mL Liquid Boluses, Performed by Patients With Chagas’ Disease (n = 17) and Controls (n = 15)

	Controls		Chagas		P value
	Mean (SD)	Median	Mean (SD)	Median	
5 mL					
OT	213 (150)	150	213 (201)	183	0.65
PT	78 (77)	50	59 (47)	51	0.72
PC	104 (70)	83	105 (185)	50	0.11
UESO	70 (62)	50	55 (39)	50	0.66
HM	220 (145)	167	119 (118)	67	0.02
OPT	269 (348)	133	256 (222)	200	0.72
10 mL					
OT	257 (183)	183	227 (227)	117	0.35
PT	58 (52)	50	77 (71)	50	0.56
PC	96 (90)	67	112 (98)	83	0.60
UESO	70 (57)	50	61 (55)	50	0.83
HM	150 (166)	50	121 (112)	67	0.92
OPT	293 (263)	167	200 (249)	83	0.11

OT: Oral transit; PT: Pharyngeal transit; PC: Pharyngeal clearance; UESO: Upper esophageal sphincter opening; HM: Hyoid movement; OPT: Oropharyngeal transit.

**Table 2:** Differences, in Milliseconds, Between Two Consecutive Swallows of 5 mL and 10 mL Paste Boluses, Performed by Patients With Chagas’ Disease (n = 17) and Controls (n = 15)

	Controls		Chagas		P value
	Mean (SD)	Median	Mean (SD)	Median	
5 mL					
OT	226 (197)	135	150 (172)	117	0.13
PT	62 (49)	50	54 (52)	33	0.54
PC	187 (228)	133	88 (79)	67	0.34
UESO	59 (44)	50	56 (73)	33	0.28
HM	173 (193)	117	328 (362)	150	0.36
OPT	241 (179)	200	271 (302)	184	0.71
10 mL					
OT	206 (133)	184	247 (152)	233	0.46
PT	34 (48)	17	70 (72)	33	0.25
PC	122 (160)	83	121 (126)	100	0.70
UESO	70 (60)	67	95 (112)	67	0.72
HM	137 (146)	117	228 (206)	200	0.09
OPT	306 (372)	167	322 (259)	317	0.52

OT: Oral transit; PT: Pharyngeal transit; PC: Pharyngeal clearance; UESO: Upper esophageal sphincter opening; HM: Hyoid movement; OPT: Oropharyngeal transit.

## Discussion

Esophageal motility disorder is an important digestive manifestation of Chagas’ disease, with alterations similar to that described in idiopathic achalasia, namely aperistaltic contraction in the esophageal body and no relaxation of the lower esophageal sphincter [2, 3].

Esophageal diseases that cause impairment of bolus transit may cause alterations of the oral and pharyngeal phases of swallowing [9, 10]. Alterations in pharyngeal transit and proximal esophageal motility, with have striated muscle and neural control by the central nervous system [11], have been described in patients with Chagas’ disease. Pharyngeal clearance is longer [4-6], and in proximal esophagus there is a late response to swallowing and a decrease in contraction amplitude, when compared with healthy subjects [12].

It is possible that the previously observed alterations in pharyngeal clearance are an adaptation to difficultly in bolus transit through the esophageal body. The involvement of the CNS is not frequent in the chronic phase of Chagas’ disease and observed alterations are not important [7, 8]. Taking into consideration previous studies of the involvement of the CNS by the disease, it is not likely that the alteration of pharyngeal clearance previously observed is consequent of this involvement. Neurons of the brain stem contain the timing pattern-generating that governs the oral, pharyngeal, and esophageal phases of swallowing [11]. The loss of the esophageal myenteric plexus caused by the disease is a more important esophageal pathological alteration [1], having consequences of esophageal motility alterations [2], characteristics of no esophageal adaptation to the bolus [13] and impairment of esophageal sensitivity [14]. However, esophageal myenteric plexus has no participation in the control of oral and pharyngeal phases of swallowing.

In Chagas’ disease, but not in healthy subjects, there is a positive correlation in pharyngeal response with the bolus volume of 5 mL and 10 mL and bolus consistency of liquid and paste. It indicates that pharyngeal clearance duration is related with the bolus characteristic in Chagas’ disease, but not in controls. The relation between pharyngeal clearance duration and hyoid movement duration is the same in patients and controls, indicating that the control of transit and hyoid movement persisted in Chagas’ disease (results not published).

It was found a lower difference between the two consecutive swallows in the hyoid movement duration after swallows of a 5 mL bolus of liquid in Chagas’ disease patients than in controls. The statistical significance of this result may be consequent of the multiple comparisons performed, which found significance in only one comparison, or to a more restrict control of hyoid movement.

In esophageal contractions there is no significant variation of amplitude and duration with successive swallows in healthy volunteers and patients with Chagas’ disease [15]. The esophageal contraction is a response of esophageal stimulation and is independent of both the oral and pharyngeal phases of swallowing [11]. In the oral and pharyngeal phases of swallowing of healthy subjects there is a substantial variability in timing of events after consecutive swallows [16, 17], a situation that our results showed to be similar in patients with Chagas’ disease.

The esophageal involvement by the disease should be treated with focus on lower esophageal sphincter, by pneumatic dilation, laparoscopic myotomy, smooth muscle relaxants, or botulinum toxin, but the results of this investigation indicated that the patients do not need treatment for alterations in the oral or pharyngeal phases of swallowing.

In conclusion, the results suggested that Chagas’ disease patients have the same central nervous system control of oral and pharyngeal phase of swallowing as healthy subjects.
